# Exocytosis of macrophage lysosomes leads to digestion of apoptotic adipocytes and foam cell formation[S]

**DOI:** 10.1194/jlr.M064089

**Published:** 2016-06

**Authors:** Abigail S. Haka, Valéria C. Barbosa-Lorenzi, Hyuek Jong Lee, Domenick J. Falcone, Clifford A. Hudis, Andrew J. Dannenberg, Frederick R. Maxfield

**Affiliations:** Departments of Biochemistry,* Weill Cornell Medical College, New York, NY 10065; Medicine,† Weill Cornell Medical College, New York, NY 10065; Pathology and Laboratory Medicine,§ Weill Cornell Medical College, New York, NY 10065; Department of Medicine,** Memorial Sloan Kettering Cancer Center, New York, NY 10065

**Keywords:** adipose tissue, obesity, lipolysis and fatty acid metabolism, extracellular catabolism, crown-like structure

## Abstract

Many types of apoptotic cells are phagocytosed and digested by macrophages. Adipocytes can be hundreds of times larger than macrophages, so they are too large to be digested by conventional phagocytic processes. The nature of the interaction between macrophages and apoptotic adipocytes has not been studied in detail. We describe a cellular process, termed exophagy, that is important for macrophage clearance of dead adipocytes and adipose tissue homeostasis. Using mouse models of obesity, human tissue, and a cell culture model, we show that macrophages form hydrolytic extracellular compartments at points of contact with dead adipocytes using local actin polymerization. These compartments are acidic and contain lysosomal enzymes delivered by exocytosis. Uptake and complete degradation of adipocyte fragments, which are released by extracellular hydrolysis, leads to macrophage foam cell formation. Exophagy-mediated foam cell formation is a highly efficient means by which macrophages internalize large amounts of lipid, which may ultimately overwhelm the metabolic capacity of the macrophage. This process provides a mechanism for degradation of objects, such as dead adipocytes, that are too large to be phagocytosed by macrophages.

Macrophage interactions with adipocytes are important both in states of metabolic dysfunction and in healthy adipose tissue expansion and remodeling (1–5). Despite this importance, our understanding of macrophage-adipocyte interactions is incomplete. It is known that adipose tissue macrophages transform into foam cells and drive the inflammatory changes that occur in adipose tissue, and it appears that macrophages play a protective role in adipose homeostasis, but mount a maladaptive immune response in the setting of obesity (6–9). Macrophages in white adipose tissue (WAT) form crown-like structures (CLSs) encircling dead or dying adipocytes (10). Recent studies, which show that adipose tissue macrophages are involved in the inflammatory changes associated with obesity, highlight the importance of understanding the biology of the macrophage-adipocyte interaction within a CLS. It is known that CLS macrophages accumulate lipid, transforming it into foam cells (8, 11). In the setting of obesity, it has been proposed that hypertrophic adipocytes release triglycerides and nonesterified fatty acids that the macrophage can then passively internalize using standard endocytic mechanisms (12). However, in this study, we show that, rather than endocytosis of released lipids, the macrophages themselves actively participate in lipid liberation from the adipocyte.

Our laboratory and others have described a process in which large moieties or species tightly bound to the extracellular matrix are initially digested by macrophages in an extracellular acidic lytic compartment (13–18). We describe this process as exophagy. We have studied exophagy in the context of macrophage degradation of aggregated LDL, as occurs during atherogenesis (14, 15). Exophagic catabolism of aggregated LDL results in uptake of cholesterol by the macrophage, leading to foam cell formation. While foam cell formation has been an area of extensive study in the atherosclerosis field, macrophage foam cell formation in CLSs has only been reported recently (11). Given the similarities between these two systems, we examined whether exophagy could be responsible for macrophage degradation of dead adipocytes. This would allow extracellular catabolism and subsequent uptake of pieces of the adipocyte, facilitating macrophage foam cell formation as a consequence of clearing dead adipocytes. Exophagy-mediated foam cell formation is a highly efficient means by which macrophages internalize large amounts of lipid, which may overwhelm the metabolic capacity of the macrophage, as has been demonstrated in the setting of atherosclerosis (19), leading to a maladaptive inflammatory response. This biology may have particular relevance during clearance of dramatically enlarged adipocytes, as occurs in the setting of obesity.

Herein, we present evidence for CLS macrophage exophagic clearance of dead adipocytes in mouse WAT. To model this biology, an in vitro CLS cell culture model was developed that mirrors several features of in vivo CLS macrophage-adipocyte interactions. Using this model, we demonstrate that CLS macrophages form an extracellular acidic hydrolytic compartment containing lysosomal enzymes delivered via exocytosis. Initial catabolism of the dead adipocyte occurs in these extracellular compartments, allowing the macrophage to internalize pieces of the adipocyte and transform it into a foam cell. We show that macrophage foam cell formation is specific to interaction with dead or dying adipocytes and is blocked when exophagy is inhibited.

## MATERIALS AND METHODS

### Animals

C57BL/6J wild-type male and female mice were purchased from Jackson Laboratory (Bar Harbor, ME). At 6 weeks of age, all male mice were randomized to receive either a low fat diet (LFD) or high fat diet (HFD) for 12 weeks. The LFD (12450Bi) and HFD (D12492i) contain 10 kcal% fat and 60 kcal% fat, respectively (Research Diets, New Brunswick, NJ) and are commonly used in studies of obesity (20). Male mice were euthanized and epididymal fat was removed and fixed with 1% formalin for immunofluorescence analysis. At 5 weeks of age, ovariectomized C57BL/6J female mice received a HFD for 10 weeks. Following euthanization, mammary fat was removed and stained for immunofluorescence or paraffin blocks were prepared for hematoxylin and eosin (H&E) staining.

### Human tissues

For each patient, paraffin blocks were prepared. Samples were examined with H&E staining.

### Cells and cell culture

Primary murine adipocytes were isolated from epididymal fat as described previously (21). Briefly, epididymal fat in DMEM/F-12 medium (Invitrogen, Carlsbad, CA) containing 1.0% BSA were chopped with surgical scissors and then digested with 0.2% collagenase type 2 (Worthington, Lakewood, NJ) for 25 min at 37°C. After passing the mixture through a 200 μm mesh filter to remove undigested fragments, the filtrate was centrifuged at 100 *g* for 10 min at 4°C. Separated adipocytes were collected with a disposable transfer pipette, washed with DMEM/F-12 medium, and attached to poly-D-lysine-coated coverslip dishes for microscopy. Coverslip dishes were functionalized with 6.15 mg/ml Bis(NHS)PEO_5_ (Pierce, Rockford, IL) in PBS (pH 9.0) for 1 h so that primary adipocytes would adhere to the dish rather than float. Dishes were then washed with HBSS, inverted, and placed on top of a cuvette containing primary adipocytes. Primary adipocytes were incubated with inverted functionalized coverslip dishes in HBSS (pH 7.8) for 1 h. To quench any unreacted *N*-hydroxysuccinimidyl ester (NHS) dishes were incubated with DMEM containing 50 mg/ml fatty acid-free BSA for 1 h. 3T3 L1 fibroblasts were cultured in DMEM supplemented with 10% calf serum, 50 units/ml penicillin, and 50 μg/ml streptomycin. Cells were differentiated into adipocytes as described previously and used 7–10 days after differentiation (22). To induce apoptosis, adipocytes were either incubated with 25 nM TNF-α for 24 h (23) or coverslip bottom dishes were exposed to 365 nm UV radiation using a 2UV Transilluminator (UVP, Upland, CA) for 1 h. To induce pyroptosis, adipocytes were incubated with 10 ng/ml lipopolysaccharide for 4 h followed by a 2 h incubation with 10 μM nigericin (24). Adipocyte death was confirmed with propidium iodide staining, performed according to the manufacturer’s protocol (Clontech, Moutainview, CA).

J774a.1 and RAW264.7 macrophage-like cells (American Type Culture Collection, Manassas, VA) were maintained in DMEM supplemented with 10% FBS, 50 units/ml penicillin, and 50 μg/ml streptomycin in a humidified atmosphere (5% CO_2_) at 37°C. Bone marrow-derived macrophages (BMMs) isolated from C57BL/6 mice were differentiated for 7 days by culture in the same medium supplemented with 20% L-929 cell-conditioned medium. Human monocytes (Life Line Cell Technology, Frederick, MD) were differentiated into macrophages in vitro by incubation in RPMI containing 10% heat-inactivated FBS and 10 ng/ml macrophage colony stimulating factor (R&D Systems, Minneapolis, MN) for 7 days. For all live cell imaging experiments, medium was changed to DMEM containing 25 mM HEPES without phenol red or sodium bicarbonate.

### Reagents

Adipocytes were labeled using succinimidyl esters of AlexaFluor (Alexa)546 and Alexa488 (Invitrogen), FITC, biotin (Sigma-Aldrich, St. Louis, MO), or CypHer 5E (GE Healthcare, Chalfont St. Giles, UK). Alexa546-biocytin, Alexa488-cholera toxin B (CtB), and LipidTOX-Red were purchased from Invitrogen. Streptavidin, bafilomycin A1, and protease inhibitor cocktail (P1860), containing aprotinin, bestatin, E-64, leupeptin, and pepstatin A, were purchased from Sigma-Aldrich.

### Tissue immunofluorescence staining

Whole-mounted epididymal fat from male mice on a LFD or HFD was incubated in 5% FBS for 1 h at room temperature for blocking after fixation with 1% formalin at 4°C for 12 h. To analyze macrophage plasma membrane lysosome-associated membrane protein-1 (LAMP-1) expression levels, murine adipose tissues were incubated with primary antibodies at 4°C for 12 h. After three washes with PBS, samples were incubated with secondary antibodies at room temperature for 4 h. For the detection of macrophage lysosomal LAMP-1 expression levels, adipose tissue was treated with 0.3% Triton for permeabilization. The primary antibodies used in this experiment were LAMP-1 (1:2,000, ab24170; Abcam, Cambridge, MA), F4/80 (1:2,000, MCA497R; AbD Serotec, Raleigh, NC), and calnexin (1:2,000, ab192439; Abcam). Anti-rabbit-cy3 (1:2,000), anti-rat-FITC (1:2,000), and anti-goat-cy5 (1:2,000) (Jackson ImmunoResearch, West Grove, PA) were used as secondary antibodies. Tissues were next stained with 1 μg/ml 4′,6-diamidino-2-phenylindole (DAPI) (Invitrogen) at room temperature for 10 min. For the detection of F-actin, epididymal fat from a male mouse on a HFD was treated with 0.3% Triton for 1 h at room temperature and then incubated with Alexa488-phalloidin (1:200, Invitrogen) at 4°C for 12 h. Images were acquired with a Zeiss LSM510 laser scanning confocal microscope using a 40× 0.8 numerical aperture (NA) objective.

Data were analyzed with MetaMorph image analysis software, Molecular Devices Corporation (Downingtown, PA). Images were convolved with a 5 × 5 pixel Gaussian filter. For LAMP-1 analysis, a binary mask was created using the FITC signal intensity, to select F4/80 positive cells, and multiplied by an inverted binary mask created using the Cy5 signal, to select calnexin-negative nonpermeabilized cells. The resultant mask was then applied to the Cy3 channel to isolate LAMP-1 signals from nonpermeabilized macrophages (F4/80 positive and calnexin negative). The surface LAMP-1 signal in each image was divided by the number of cells in that field, as determined by DAPI staining, to generate an average value for plasma membrane LAMP-1 per cell. For permeabilized tissues, a binary mask was created using the FITC signal intensity to select F4/80 positive cells. This mask was applied to the Cy3 channel to isolate LAMP-1 signals from macrophages. The LAMP-1 signal was divided by the number of cells in each image, as determined by DAPI staining, to calculate a value for lysosomal LAMP-1 per macrophage.

### Cell labeling and microscopy

Plasma membrane labeling with Alexa488-CtB (15) and F-actin labeling with Alexa488-phalloidin (14) were performed as described previously. Plasma membrane labeling with Alex488-CtB was either followed by fixation in 3% paraformaldehyde (PFA) for 15 min (to allow the CtB on its lipid receptor to redistribute into the sealed zone between the adipocyte and macrophage following fixation) or 3% PFA and 0.5% glutaraldehyde for 30 min (to avidly cross-link plasma membrane proteins preventing postfixation diffusion of the CtB on its lipid receptor). Apoptotic adipocytes were labeled with various fluorophores or biotin conjugated to succinimidyl esters by incubation of the cells with 0.02 mg/ml of the conjugated fluorophore in DMEM for 1 h. Streptavidin labeling of adipocytes was accomplished by incubating biotin-labeled cells with 0.5 mg/ml streptavidin in DMEM for 1 h. To determine surface LAMP-1 expression in the CLS cell culture model, J774 cells were incubated with apoptotic adipocytes or live adipocytes, fixed without permeabilization in 1% PFA for 5 min, and incubated in PBS with 10% goat serum for 1 h. Next, cells were labeled with an antibody against LAMP-1 (Abcam) at 1:125 dilution in the presence of 3% goat serum for 60 min, washed, and incubated with anti-rabbit-Alexa488 at 1:250 dilution for 30 min. An irrelevant antibody was used as a control for LAMP-1 immunostaining. Surface LAMP-1 imaging was carried out with the pinholes open (to improve signal collection) resulting in an axial resolution of 14 μm.

For imaging, cells were plated on poly-D-lysine-coated glass coverslip bottom dishes. Images were acquired with a Zeiss LSM510 laser scanning confocal microscope using either a 40× 0.8 NA plan Apochromat objective or a 63× 1.4 NA plan Apochromat objective.

### Lysosome exocytosis

Lysosome labeling of macrophages plated in a tri-partitioned Petri dish was accomplished via an 18 h pulse with 2.2 mg/ml biotin-fluorescein-dextran. Cells were chased for 4 h in DMEM and subsequently incubated with apoptotic streptavidin-labeled adipocytes for 90 min. Labeling of extracellular streptavidin-conjugated adipocytes was accomplished with a 30 s pulse of 200 μM Alexa546-biocytin at the end of the 90 min incubation. Cells were incubated with 200 μM biotin for 10 min in order to bind any unoccupied streptavidin sites prior to cell permeabilization. Cells were then fixed with 3% PFA for 15 min, washed, and permeabilized with 1% Triton for 10 min. Images were acquired with a Zeiss LSM 510 laser scanning confocal microscope using a 40× 0.8 NA objective.

### pH measurements

Macrophages were incubated for 60 min with CypHer 5E (a pH sensitive fluorophore) and Alexa488 (a pH insensitive fluorophore) dual-labeled apoptotic adipocytes. The pH value within each pixel was assessed quantitatively by comparison with ratio images obtained in calibration buffers of varying pH, as described previously (15). Nuclear regions were excluded from the calibration curve, as the two dyes accumulated at different rates within the nucleus. Cell temperature was maintained at 37°C with a heated stage.

All data were analyzed with MetaMorph image analysis software, Molecular Devices Corporation. A binary mask was created using the Alexa488 signal intensity and applied to both channels to remove background noise. Images were convolved with a 7 × 7 pixel Gaussian filter, and ratio images were generated.

### Adipocyte internalization

J774 cells were incubated with Alexa546-labeled TNF-α-induced apoptotic primary murine adipocytes for 90 min in the presence or absence of 2 μM bafilomycin A1. The macrophage plasma membrane was labeled with Alexa488-CtB, as described above, and samples were fixed with 1% PFA for 15 min. The Alexa546 signal in each macrophage touching an adipocyte was quantified. We note that the coculture system often contains small amounts of cellular debris from the apoptotic adipocytes. Macrophages may internalize this debris using endocytic mechanisms other than exophagy. To minimize the effects of adiopcyte cellular debris on our results, Alexa546 signal was only quantified in those macrophages in contact with an adipocyte. The cells in each image were identified using Alexa488-CtB cell surface staining. The fluorescence power was then calculated as the sum of all the pixel intensities within the cell boundaries. As a control, J774 cells were incubated with Fluoresbrite YG latex beads (Polysciences, Inc., Warrington, PA) for 90 min in the presence or absence of 2 μM bafilomycin A1.

### Electron microscopy

RAW264.7 cells were incubated with apoptotic 3T3 L1 adipocytes or primary murine adipocytes for 90 min. For transmission electron microscopy (EM), the cells were fixed with a modified Karnovsky’s solution containing 2.5% glutaraldehyde, 4% PFA, and 0.02% picric acid, postfixed with 1% osmium tetroxide, 1.5% potassium ferricyanide, treated with uranyl acetate, dehydrated through a graded ethanol series, and embedded in LX112 resin. En face serial sections were cut at 70 nm thickness and picked up on formvar-coated 4-slot copper grids. Sections were further contrasted with uranyl acetate and lead citrate. Images were acquired at Weill Cornell Medical College on a JEOL JEM 100CX-II electron microscope operating at 80 kV. For 3D focused-ion beam scanning EM (FIB-SEM), the resin block was attached to a scanning EM stub with double-sided carbon tape and painted with colloidal silver. It was then coated with a thin layer of Au/Pd in an Ar environment (25 mA current, 60 mtorr, 5 min). The sample was loaded into an FEI Helios Nanolab 650 FIB-SEM for ion-abrasion tomography. An area of interest was found and coated with 1 μm Pt using the gas injection system of the microscope. A trench was dug in front of the area using the ion beam. Imaging of the cross-sectional face was done at an electron beam viewing angle of −30 degrees. The FEI Slice and View G2 programs were used to collect serial images using the following parameters: working distance, 2.4 mm; landing energy, 2 keV; current, 200 pA; horizontal field width, 41 μm; dwell time, 1 μs; 4× integration; 4096 pixels across (10 nm/pixel); 20 nm slice width; 1,500 images. Images were stacked into a single file, shrunk by a factor of 2 in X and Y then aligned using IMOD tools. The aligned stack was imported into Imaris (Bitplane, Oxford Instruments, Concord, MA) for segmentation and analysis. Sealed compartments contained no gap in electron density between the macrophage and apoptotic adipocytes (resolution 10 nm).

### Foam cell formation

J774 cells were incubated with primary murine apoptotic adipocytes for 24 h in the presence or absence of a protease inhibitor cocktail used at a 1:800 dilution. Samples were fixed with 1% PFA for 15 min and then labeled with LipidTOX-Red according to the manufacturer’s protocol. The LipidTOX signal in each macrophage touching an adipocyte was calculated as the sum of all the pixel intensities within the cell boundaries. As a control, J774 cells were incubated with Fluoresbrite YG latex beads (Polysciences, Inc.) for 90 min in the presence or absence of protease inhibitor cocktail used at a 1:800 dilution.

To examine foam cell formation in the presence of live or dead adipocytes, J774 cells were incubated with untreated 3T3 L1 adipocytes or TNF-α-induced apoptotic 3T3 L1 adipocytes for 24 h. The macrophage plasma membrane was labeled with Alexa488-CtB as described above. Samples were fixed with 1% PFA for 15 min, labeled with LipidTOX-Red according to the manufacturer’s protocol, and the LipidTOX intensity per macrophage touching an adipocyte quantified.

### Statistics

Statistical analysis was performed using Matlab R2012A (Mathworks, Natick, MA). Data acquired on different days were compared by normalizing the data from each day to the median control value for that day. Macrophage response to apoptotic adipocytes is heterogeneous, with some cells responding vigorously and others not at all. This results in a nonnormal distribution of the macrophage response. Thus, for such comparisons of two groups, a Wilcoxon rank sum test was used. In experiments where the data was normally distributed, a Student’s *t*-test was performed.

### Study approval

The animal protocol was approved by the Institutional Animal Care and Use Committee at Weill Cornell Medical College (New York, NY). Studies involving human tissue were approved by the Institutional Review Boards of Memorial Sloan-Kettering Cancer Center and Weill Cornell Medical College. Women undergoing mastectomy at Memorial Sloan-Kettering Cancer Center were consented under a standard tissue acquisition protocol.

## RESULTS

### CLS macrophages in murine WAT exhibit increased surface LAMP-1 indicative of lysosome exocytosis

**Fig. 1.**
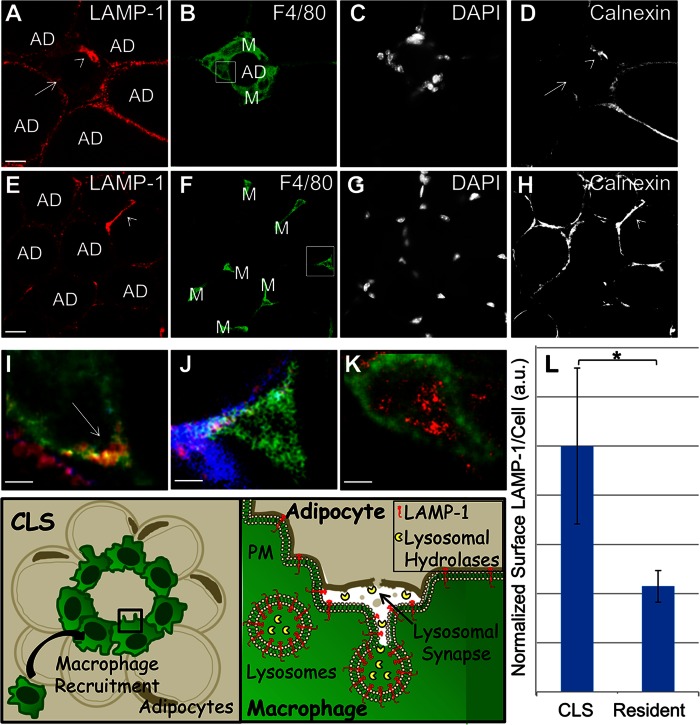
CLS macrophages in murine WAT exhibit increased plasma membrane LAMP-1 staining. A–D: WAT from mice on the HFD was labeled with LAMP-1 (A); F4/80 to indicate macrophages (B); the nuclear stain, DAPI (C); and the endoplasmic reticulum-marker, calnexin, to identify permeable cells (D). E–H: WAT from mice on the LFD was also labeled with LAMP-1 (E), F4/80 (F), DAPI (G), and calnexin (H). Size bars 20 μm. I, J: Enlarged views of representative macrophages for each condition highlighted by boxes in the F4/80 images (red, LAMP-1; green, F4/80; blue, calnexin). A nonpermeable CLS macrophage demonstrating plasma membrane LAMP-1 (arrow) (I) and a nonpermeable resident macrophage that is negative for plasma membrane LAMP-1 (J). K: A macrophage from detergent-permeabilized tissue demonstrating lysosomal LAMP-1, included for comparison. Size bars 5 μm. A total of ten mice were examined, five on the HFD and five on the LFD. L: Plasma membrane LAMP-1 levels were quantified from 1,466 WAT macrophages in mice on the HFD or LFD. Macrophages that were permeable, as assessed by calnexin staining, were excluded from the analysis [e.g., arrowheads in (A) and (D), and (E) and (H)]. Surface LAMP-1 staining more than doubled in CLS macrophages compared with resident macrophages (L). Error bar represents the SEM. *P ≤ 0.05 Wilcoxon rank sum test. Representative adipocytes (AD) and macrophages (M) are labeled. A schematic of the biology under investigation and experimental approach to assay lysosome exocytosis is included.

To see whether exophagy occurs during macrophage interactions with dead adipocytes, we first examined the amount of lysosome exocytosis in CLSs and resident macrophages in an established mouse model of obesity. As a marker of lysosome exocytosis, we quantified macrophage plasma membrane LAMP-1 levels. LAMP-1 on the surface of cells is normally at extremely low levels (25, 26), and surface LAMP-1 expression has been shown to be a marker of fusion of lysosomes with the plasma membrane (27). An antibody against F4/80, a specific cell-surface marker for murine macrophages, was used to identify macrophages in WAT from mice on the HFD or the LFD, and their surface and total LAMP-1 expression were measured. DAPI was used to quantify the number of cells present, and calnexin (an endoplasmic reticulum marker) was used to examine cell permeability. A schematic of the biology under investigation and experimental approach to investigate lysosome exocytosis is included for clarity (**Fig. 1**).

Figure 1A–D shows CLS macrophages in WAT from a mouse on the HFD, while Fig. 1E–H shows resident macrophages in WAT from a mouse on the LFD. Macrophages (M) are the small F4/80-labeled cells (green) surrounding (CLS) or interspersed with (resident) adipocytes (black, AD). To ensure that LAMP-1 signals emanated from the plasma membrane and not the lysosomes, permeable macrophages were excluded from the analysis. Permeable macrophages (e.g., arrowhead, Fig. 1D, H) were identified by labeling with antibody to calnexin (an endoplasmic reticulum marker). We found that 22% of macrophages in the HFD samples and 37% of macrophages in the LFD samples were positive for calnexin.

Co-localization of the F4/80 (green) and LAMP-1 (red) staining demonstrates LAMP-1 on the plasma membrane of CLS macrophages (e.g., arrow, Fig. 1I). LAMP-1 staining is not observed on the surface of a macrophage in a mouse on the LFD (Fig. 1J). For comparison, a macrophage in detergent-permeabilized WAT showing LAMP-1 labeling of lysosomes is shown in Fig. 1K. Nonpermeabilized macrophages, as assessed by positive F4/80 and negative calnexin staining, were used to quantify macrophage plasma membrane LAMP-1 levels. We made measurements on 1,047 macrophages from five mice on the HFD and on 419 macrophages from five mice on the LFD. DAPI and F4/80 labeling was used to quantify the number of macrophages present. Supplementary Fig. 1 displays a schematic of the data analysis procedure. Figure 1L shows the amount of plasma membrane LAMP-1 staining per cell in CLS macrophages from mice on the HFD and resident macrophages from mice on the LFD. Surface LAMP-1 staining more than doubled in CLS macrophages compared with resident macrophages. These data indicate that, in vivo, CLS macrophages undergo lysosome exocytosis.

It has been reported that obesity induces a program of lysosome biogenesis in adipose tissue macrophages (28). In light of this, we examined the abundance of lysosomal proteins in our system. We labeled LAMP-1 in detergent-permeabilized WAT from mice on the HFD and the LFD. No statistically significant difference in lysosomal LAMP-1 was found (supplementary Fig. 2A–G).

### CLS cell culture model recapitulates features of in vivo CLSs

The increase in surface LAMP-1 in CLS macrophages suggests that macrophage lysosome exocytosis is occurring in vivo in a mouse model of obesity. To further investigate macrophage interactions with dead adipocytes, we developed a model system to study aspects of the interplay that are challenging to document in vivo. In this model, adipocyte death was induced by either TNF-α or UV radiation. TNF-α is a pro-inflammatory cytokine that is increased in obese WAT and induces adipocyte apoptosis in more than 50% of the cells after 24 h of incubation (23, 29). We note that the mechanism of adipocyte death in vivo is controversial and other forms of cell death, such as pyroptosis, may play a role in addition to apoptosis (12, 30, 31). Thus, we also replicated select experiments using pyroptotic adipocytes. Primary murine adipocytes or fully differentiated 3T3 L1 adipocytes were induced to undergo apoptosis and then incubated with either primary macro­phages or macrophage cell lines to create an in vitro CLS. Under our conditions, we observed that 90% of UV-treated adipocytes were positive for propidium iodide nuclear labeling, and 70% of adipocytes treated with TNF-α were propidium iodide positive. We also observed that 60% of the propidium iodide-negative adipocytes irradiated with UV light became labeled with annexin V and 45% for adipocytes treated with TNF-α, indicating that they expressed phosphatidylserine on the cell surface and were undergoing cell death.

**Fig. 2.**
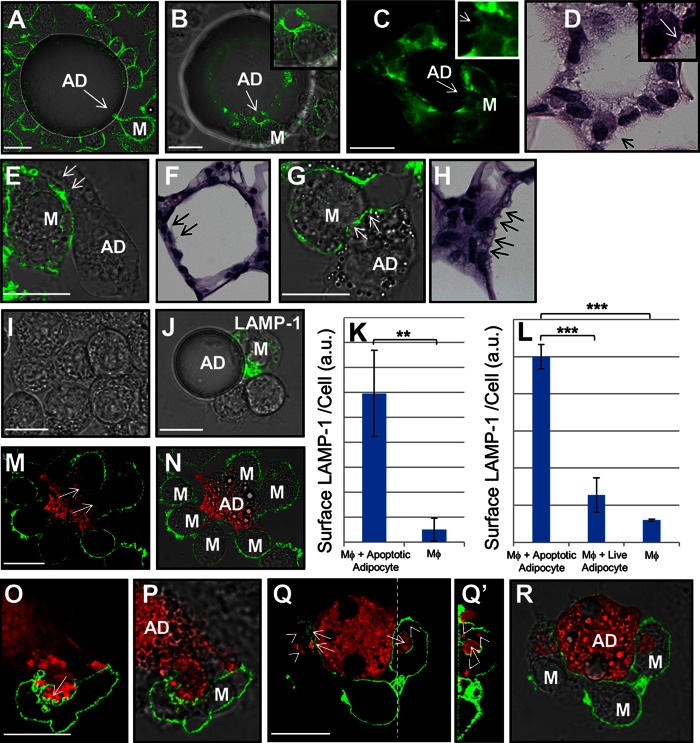
CLS cell culture model mirrors in vivo CLS morphology and shows formation of extracellular compartments at sites of contact with apoptotic adipocytes. A: An in vitro CLS in which J774 cells had their plasma membrane labeled with Alexa488-CtB (green) was incubated with UV-induced apoptotic primary murine adipocytes. A macrophage is seen extending plasma membrane protrusions at the point of contact with the adipocyte [arrow (A)]. B: An in vitro CLS in which J774 cells were incubated with UV-induced apoptotic adipocytes and labeled with Alexa488-phalloidin to show F-actin (green). The macrophage, highlighted by an arrow and shown in the inset, is extending F-actin-rich protrusions at the site of contact with the adipocyte. C: F-actin-rich macrophage protrusions (arrow and inset) are also observed in inflamed WAT from a mouse. D: H&E image of a CLS in human breast. The macrophage highlighted by an arrow and shown in detail in the inset can be seen extending protrusions similar to those observed in the model system (B). E: An in vitro CLS in which a macrophage is seen extending F-actin-rich processes at the site of contact with an UV-induced apoptotic adipocyte (arrows). F: Similar processes are seen in an H&E image of a CLS in murine WAT (arrows). G: An in vitro CLS in which the macrophage forms an F-actin-scalloped border at points of contact with an UV-induced apoptotic adipocyte (arrows). H: CLS macrophages in an H&E-stained section of murine mammary WAT exhibit membrane scalloping (arrows). I–K: J774 cells were incubated with UV-induced apoptotic primary murine adipocytes for 90 min. I: In control macrophages, no LAMP-1 immunostaining (green) was observed on the plasma membrane. J: Upon incubation with apoptotic adipocytes, macrophage plasma membrane LAMP-1 (green), a marker of lysosome exocytosis, was increased. K: Quantification of the macrophage surface levels of LAMP-1. Data are from 201 cells in one experiment. Error bar represents the SEM. **P ≤ 0.005 Wilcoxon rank sum test. L: Quantification of the macrophage surface levels of LAMP-1 after incubation with either TNF-α-induced apoptotic or viable 3T3 L1 adipocytes. Macrophage plasma membrane LAMP-1 level was strongly reduced in macrophages incubated with viable adipocytes compared with macrophages incubated with apoptotic adipocytes. Data are from 301 cells in three independent experiments. Error bar represents the SEM. ***P ≤ 0.001 Wilcoxon rank sum test. M–R: Macrophages form extracellular compartments at sites of contact with UV-induced apoptotic adipocytes. 3T3 L1 apoptotic adipocytes were labeled with Alexa546 (red) and incubated with J774 cells for 60 min. Cells were then labeled with Alexa488-CtB on ice and fixed. Representative images are of apoptotic adipocytes (red) and macrophages (green). Fixation that cross-links plasma membrane proteins, preventing postfixation diffusion of the CtB, reveals large sealed zones at the macrophage-adipocyte interface [arrows (M)]. Fixation that allows postfixation diffusion of the CtB on its lipid receptor into the sealed zones reveals extensive plasma membrane ruffling in the region of contact between the macrophage and apoptotic adipocyte [arrow (O)]. Pieces of the adipocyte are seen in extracellular compartments, surrounded by plasma membrane [arrows (Q)], as well as in completely internalized vesicles [arrowheads (Q)], which are negative for CtB. Q’: A confocal vertical section through the dashed line in Q. Size bars 20 μm. AD, adipocyte; M, macrophage.

**Figure 2A** displays an image of an in vitro CLS. In this experiment, UV-induced apoptotic primary murine adipocytes were incubated with J774 cells for 60 min. The macrophage plasma membrane was labeled with Alexa488-CtB, as described previously (15). A macrophage is seen extending plasma membrane protrusions at the point of contact with the dead adipocyte (arrow, Fig. 2A). Figure 2B shows an image of an in vitro CLS labeled with Alexa488-phalloidin to show F-actin. Actin polymerization is important in the formation of the exophagic compartment used to digest aggregated LDL during macrophage foam cell formation in atherosclerosis (14, 32). The macrophage highlighted by an arrow in Fig. 2B, and shown in detail in the inset, is seen extending F-actin-rich protrusions at the site of contact with the UV-induced apoptotic adipocyte.

Several interesting morphologic features observed at the macrophage-adipocyte interface in the model system were also observed in CLSs in human and murine tissues. Confocal imaging of a CLS in murine WAT reveals actin-rich macrophage protrusions extending toward an adipocyte (arrow and inset, Fig. 2C) very similar to the protrusions seen in vitro (Fig. 2B). Fig. 2D shows a H&E image of a CLS in WAT from human breast. The fine ruffling of the macrophage membranes at the macrophage-adipocyte interface is created by numerous cytoplasmic extensions. Macrophage protrusions, similar in morphology to those seen in the model system (Fig. 2B), are highlighted by an arrow and shown in the inset in detail.

Additional examples of morphologic similarities between in vitro and in vivo CLSs are shown in Fig. 2E–H. J774 cells were incubated with UV-induced apoptotic 3T3 L1 adipocytes for 60 min, fixed, and stained with Alexa488-phalloidin. In Fig. 2E, macrophages in the model system are seen extending cellular processes containing F-actin toward an apoptotic adipocyte. Similar protrusions are seen in inflamed murine WAT (Fig. 2F). Scalloping of the macrophage plasma membrane is observed at the macrophage-apoptotic adipocyte interface in the model system (arrows, Fig. 2G) and in inflamed murine WAT (arrows, Fig. 2H).

Next, we investigated whether the CLS cell culture model showed increases in macrophage plasma membrane LAMP-1, similar to those observed in murine tissues. To test this, we performed immunofluorescence surface labeling of LAMP-1 in nonpermeabilized J774 cells incubated with UV-induced apoptotic primary murine or 3T3 L1 adipocytes. In control J774, very little or no basal surface expression of LAMP-1 was observed (Fig. 2I). Upon incubation with primary murine apoptotic adipocytes for 90 min, the macrophage plasma membrane LAMP-1 staining was significantly increased (Fig. 2J, K). Also, when macrophages were incubated with viable 3T3 L1 adipocytes, a low surface expression of LAMP-1 was observed compared with macrophages in the presence of TNF-α apoptotic adipocytes (Fig. 2L). The increase in plasma membrane LAMP-1 was recapitulated in human monocyte-derived macrophages (huMDMs) incubated with UV-induced apoptotic primary murine adipocytes (supplementary Fig. 2H). Immunostaining was carried out with an irrelevant antibody as control for the LAMP-1 antibody (supplementary Fig. 3). These cell culture results are consistent with the LAMP-1 observations in murine WAT tissue (Fig. 1).

### Macrophages form specialized extracellular compartments at sites of contact with apoptotic adipocytes

The morphological similarities between in vivo CLSs and in vitro CLSs supports the relevance of the model system. Thus, we used the model system to investigate aspects of CLS biology that are challenging to document in vivo. To visualize the macrophage-adipocyte interface in greater detail, UV-induced apoptotic 3T3 L1 adipocytes were labeled with Alexa546 and incubated with J774 cells for 60 min. Cells were then labeled with Alexa488-CtB on ice for 2 min, washed, and fixed. Alexa488-CtB was excluded from sites of contact between the macrophage and adipocyte, indicating a sealed zone between the two cells (arrows, Fig. 2M, N). To visualize the topological organization of the sealed zone between the macrophage and adipocyte, the Alexa488-CtB was allowed to redistribute on the plasma membrane following fixation. It is known that glycosylphosphatidylinositol (GPI)-anchored proteins can redistribute if the fixative does not avidly cross-link the receptor to the plasma membrane (33), so we anticipated that CtB bound to gangliosides would also be able to redistribute after mild fixation with formaldehyde. Using this approach, we observed that sites of contact between the macrophage and adipocyte were complex surfaces containing smaller sub-compartments within the sealed zone (Fig. 2O, P). Plasma membrane ruffling (arrow, Fig. 2O), reminiscent of the ruffled border seen during osteoclast bone resorption (17), was seen.

Following a 60 min incubation, pieces of the adipocyte (red) were seen in extracellular compartments, surrounded by macrophage plasma membrane (green) (arrows, Fig. 2Q, R). Figure 2Q’, a 3D confocal reconstruction through the dashed line in Fig. 2Q, shows a large piece of adipocyte (arrow, Fig. 2Q’) that is sequestered from the main body of the adipocyte and contained in an extracellular compartment. Adipocyte fragments are also visible in completely internalized vesicles (arrowheads, Fig. 2Q, Q’) that are negative for CtB labeling. The ability of the macro­phage to internalize pieces of the adipocyte is suggestive of extracellular catabolism, which would allow the macrophage to break off partially digested pieces of the adipocyte.

### Macrophage lysosome exocytosis occurs in CLSs

In order for adipocytes to undergo catabolism extracellularly, macrophages must secrete a hydrolase. Our LAMP-1 data indicates that lysosome exocytosis occurs in CLS macrophages both in vivo and in the in vitro model system. We tested whether macrophage lysosomal contents were delivered to extracellular sites of macrophage interaction with dead adipocytes. Biotin-fluorescein-dextran was incubated with murine BMMs overnight, leading to endocytosis of the dextran and delivery to lysosomes (15). Apoptotic adipocytes were labeled with a succinimidyl ester conjugate of biotin and then incubated with streptavidin. BMMs with biotin-fluorescein-dextran-loaded lysosomes were incubated with streptavidin-labeled apoptotic adipocytes for 90 min. Lysosome exocytosis is detected by binding of the biotin-fluorescein-dextran to the streptavidin labeled adipocyte. At the end of the experiment, a 30 s treatment with biotin-Alexa546 was used to label extracellular streptavidin on the adipocyte. [We have previously shown that the extracellular compartments formed by macrophages are dynamic, allowing catabolic products and lysosomal enzymes to be released into the extracellular space as well as the entry of molecules such as biotinylated fluorophores (15).] Next, excess unlabeled biotin was applied to block any remaining free streptavidin on the adipocytes. Cells were then fixed and permeabilized.

**Fig. 3.**
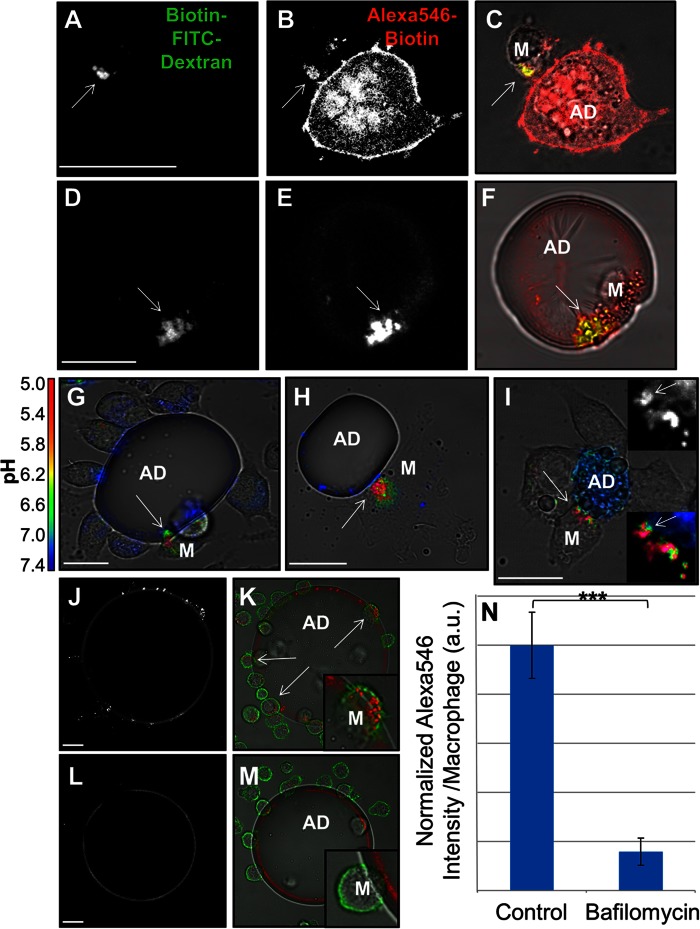
Lysosome exocytosis and acidification in extracellular compartments formed at sites of contact with apoptotic adipocytes. J774 cells (A–C) or huMDMs (D–F) were incubated overnight with biotin-fluorescein-dextran to deliver the dextran to lysosomes. Cells were then incubated with streptavidin-labeled UV-induced apoptotic 3T3 L1 adipocytes (A–C) or UV-induced apoptotic primary murine adipocytes (D–F) for 90 min followed by a 30 sec treatment with 200 μM biotin-Alexa546 to mark extracellular streptavidin-labeled structures. Cells were then fixed and permeabilized to remove unbound biotin-fluorescein-dextran from the lysosomes. Representative images of biotin-fluorescein-dextran (A, D), biotin-Alexa546 (B, E), and merged images superimposed on phase contrast images (C, F). Colocalization of the fluorescein and Alexa546 signals demonstrates that lysosomal contents are delivered to extracellular compartments (arrows). G–I: Macrophages were incubated with apoptotic adipocytes labeled with a pH-sensitive fluorophore and a pH-insensitive fluorophore. Ratiometric images showing acidified compartments as J774 cells interact with a TNF-α-induced apoptotic primary murine adipocyte (G), a huMDM interacts with a TNF-α-induced apoptotic primary murine adipocyte (H), and J774 cells interact with an UV-induced apoptotic 3T3 L1 adipocyte (I). Regions of low pH are contiguous with the adipocyte body, thus supporting that they are extracellular [insets, arrows (I)]. The lower inset is an enlarged image of the macrophage-adipocyte interface showing variations in pH. The upper inset shows the CypHer fluorescence of the same region as the lower inset and is included for clarity. J–N: TNF-α-induced apoptotic primary murine adipocytes were labeled with Alexa546 and incubated with J774 cells for 90 min in the absence (J, K) or presence (L, M) of bafilomycin A1, a V-ATPase inhibitor. Following the 90 min incubation, macrophages were labeled with Alexa488-CtB on ice, and the amount of Alexa546 signal within the macrophage was quantified. N: Bafilomycin A1 treatment abolished macrophage adipocyte uptake. Data are from three independent experiments. Error bars represent the SEM. ***P ≤ 0.001 Wilcoxon rank sum test. Size bars 20 μm. AD, adipocyte; M, macrophage.

When BMMs (**Fig. 3A–C**) or huMDMs (Fig. 3D, F) were in contact with UV-induced apoptotic adipocytes for 90 min, there was significant deposition of previously internalized biotin-fluorescein-dextran (Fig. 3A, D) onto the adipocyte in regions in contact with the macrophage. Colocalization of the fluorescein and biotin-Alexa546 signals (arrows, Fig. 3A–F) clearly indicates that lysosomal contents were extracellular and delivered to the macrophage-adipocyte interface. While we display compartments that are positive for Alexa546, we note that many regions of exocytosis excluded biotin-Alexa546 (supplementary Fig. 3). These may represent areas of the compartment that are transiently sealed sufficiently to hold a pH gradient. To investigate the effects of mechanisms of adipocyte death other than apoptosis, 3T3 L1 fully differentiated adipocytes were treated with nigericin to induce pyroptosis. Lysosome exocytosis was observed when RAW264.7 macrophages were incubated with pyroptotic adipocytes (supplementary Fig. 4A–C), indicating that macrophage exophagy occurs with other mechanisms of adipocyte death. To address whether lysosome exocytosis occurs exclusively during the interaction of macrophages with apoptotic adipocytes, we investigated lysosomal synapse formation in J774 macrophages incubated with viable 3T3 L1 adipocytes. The deposition of lysosomal biotin-fluorescein-dextran on either TNF-α apoptotic or live adipocytes was quantified. About five times more biotin-fluorescein-dextran was detected on the surface of apoptotic adipocytes, as compared with the living adipocytes (supplementary Fig. 4D–F). The vast majority of live adipocytes that had their surface labeled with NHS-biotin and streptavidin remained viable, as assessed by propidium iodide staining (supplementary Fig. 4G). These data show that macrophage lysosomal contents were secreted upon contact with dead adipocytes, thereby providing hydrolases for extracellular adipocyte catabolism.

### The macrophage-apoptotic adipocyte interface is acidified

An acidic environment is required for secreted lysosomal acid hydrolases to function. To test whether the macrophage-adipocyte interface is acidified, we labeled apoptotic adipocytes with CypHer 5E, a pH-sensitive fluorophore (34), and Alexa488, a pH-insensitive fluorophore. Macrophages were incubated with dual-labeled adipocytes, and the pH at points of cell contact was determined by ratiometric live cell imaging. When J774 cells (Fig. 3G) or huMDMs (Fig. 3H) interacted with TNF-α-induced apoptotic primary murine adipocytes, regions of low pH, sufficient for activation of most lysosomal acid hydrolases, could be seen at the contact sites (arrows, Fig. 3G, H). Similar results were obtained using 3T3 L1 UV-induced apoptotic adipocytes and J774 cells (Fig. 3I). Regions of low pH are contiguous with the apoptotic adipocyte body, thus supporting that they are extracellular (insets, arrows, Fig. 3I). The acidic regions are transient, dissipating, and reforming on the timescale of minutes. This can be observed in time-lapse ratiometric live cell imaging (supplementary Movie 2) and indicates that compartments formed for exophagy can be tightly sealed at times, but also transiently open. Acidified surface compartments were not seen in J774 cells incubated with live 3T3 L1 adipocytes (supplementary Fig. 4H; supplementary Movie 1). These data show that compartments formed at the apoptotic adipocyte-macrophage interface contain lysosomal enzymes and are acidified, thereby allowing their activity. These compartments can be considered a lysosomal synapse.

We have shown previously that lysosomal synapse acidification depends on the activity of the plasma membrane vacuolar (H^+^)-ATPase (V-ATPase) (15), which translocates protons from the cytoplasm to the extracellular space (35). To test the effect of compartment pH neutralization on macrophage exophagy, TNF-α-induced apoptotic primary murine adipocytes were labeled with Alexa546 and incubated with J774 cells for 90 min in the absence (Fig. 3J, K) or presence (Fig. 3L, M) of bafilomycin A1, a V-ATPase inhibitor (36). Following the 90 min incubation, macrophages were labeled with Alexa488-CtB for 2 min on ice, and the amount of Alexa546 signal within the macrophages was quantified. Bafilomycin A1 treatment almost completely abolished adipocyte uptake by macrophages (Fig. 3N). These data demonstrate that V-ATPase in the macrophage plasma membrane is responsible for the acidification of the lysosomal synapse formed during macrophage exophagy. This acidification enables adipocyte hydrolysis by lysosomal enzymes and subsequent macrophage degradation and internalization of pieces of the dead adipocyte. Bafilomycin A1 will also prevent degradation in lysosomes, so material taken into bafilomycin A1-treated cells should remain undigested. As a control, phagocytosis of fluorescently labeled beads was quantified in J774 cells in the presence or absence of bafilomycin A1 (supplementary Fig. 5A). Bafilomycin A1 treatment had no effect on phagocytosis (supplementary Fig. 5A). This shows that the effect caused by bafilomycin A1 is due to inhibition of exophagy-mediated uptake.

### Visualization of extracellular compartments at the macrophage-apoptotic adipocyte interface that can hold a pH gradient

**Fig. 4.**
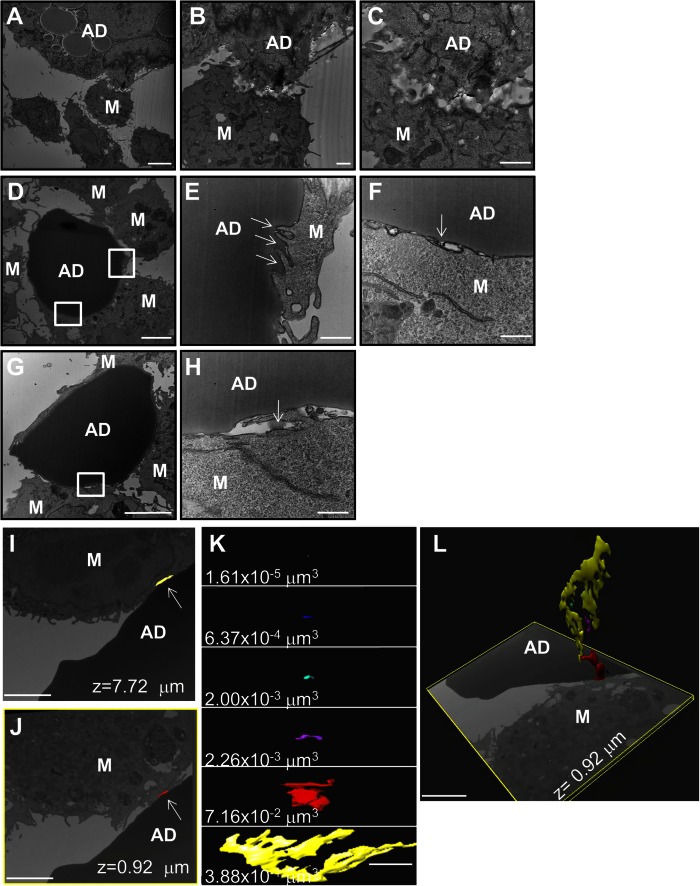
Visualization of extracellular compartments at the macrophage-adipocyte interface that are able to hold a pH gradient. A–H: EM images acquired to investigate the macrophage-apoptotic adipocyte interaction. A–C: RAW264.7 cells were incubated with TNF-α-induced apoptotic 3T3 L1 adipocytes for 90 min. Macrophage plasma membrane ruffling and lacunae formation are observed at sites of contact between the macrophage and dead adipocyte. D–H: EM images of RAW264.7 cells interacting with TNF-α-induced apoptotic primary murine adipocytes. E: A macrophage, highlighted by the upper box in (D), can be seen penetrating the adipocyte body and sequestering pieces of the adipocyte at points of contact (arrows). F: A macrophage, highlighted by the lower box in (D), is observed digesting the adipocyte plasma membrane in an extracellular compartment (arrow). G, H: Material with an electron density similar to the adipocyte can be seen in an extracellular compartment [arrow (H)], highlighted by a box in (G). Size bars 5 μm (A ,D, G), 1 μm (B, C), 500 nm (E, F, H). I–L: A FIB-SEM data set showing several sealed compartments at the macrophage apoptotic-adipocyte interface. I: A sealed compartment is shown in yellow (arrow) in a single z-slice. J: A compartment formed at a different z-position is highlighted in red (arrow). K: Individual reconstructed compartments are shown with their corresponding volume. L: Sealed extracellular compartments are displayed in 3D with a single z-slice at the same position as the image shown in (J). Size bars 2 μm. AD, adipocyte; M, macrophage.

To examine the compartment formed at the macrophage-adipocyte interface in greater detail, in vitro CLSs were imaged by transmission EM and FIB-SEM. 3T3 L1 adipocytes were induced to undergo apoptosis by incubation with TNF-α. Adipocytes were washed thoroughly to remove any TNF-α and incubated with RAW264.7 cells for 90 min. Macrophage plasma membrane ruffling and lacunae formation were observed at sites of contact between the macrophage and dead adipocyte (**Fig. 4A–C**). The apparent absence of adipocyte plasma membrane in the region of contact between the macrophage and dead adipocyte is indicative of extracellular catabolism (Fig. 4C). Figure 4D–H shows EM images of RAW264.7 cells interacting with apoptotic primary murine adipocytes. In a region highlighted by the upper box in Fig. 4D, a macrophage is seen sequestering pieces of the adipocyte at points of contact (arrows, Fig. 4E). An adjacent macrophage, highlighted by the lower box in Fig. 4D, appears to pull the adipocyte plasma membrane away from the interior lipid droplet (arrow, Fig. 4F). Most importantly, material with an electron density similar to the adipocyte’s lipid core can be seen in an extracellular compartment, highlighted by a box in Fig. 4G (arrow, Fig. 4H).

We used FIB-SEM to visualize compartments at the macro­phage-apoptotic adipocyte interface in 3D with high resolution to verify that they were sealed and would be able to retain a proton gradient. Single slices from a FIB-SEM data set acquired at different depths are shown in Fig. 4I, J. Several distinct compartments that were sealed in all dimensions were formed at the macrophage-apoptotic adipocyte interface. Each compartment was reconstructed using Imaris software and is displayed in a different color. Figure 4I shows a sealed compartment, highlighted in yellow, in a single z-section, while Fig. 4J displays a distinct compartment, highlighted in red, formed at a different z-position. Supplementary Movie 3 contains the entire FIB-SEM z-stack with the reconstructed compartments overlaid in their respective colors on each xy plane. Each individual sealed compartment at the macrophage-apoptotic adipocyte interface and its corresponding volume are shown in Fig. 4K. The 3D reconstructions of the compartments are displayed in Fig. 4L along with a single z-slice at the same position as the image shown in Fig. 4J. Supplementary Movie 4 shows the 3D reconstruction of the compartments as the FIB-SEM images are varied in the z dimension followed by examination of both cells and compartments in 3D.

### Macrophage exophagy leads to foam cell formation and can be blocked with protease inhibition

**Fig. 5.**
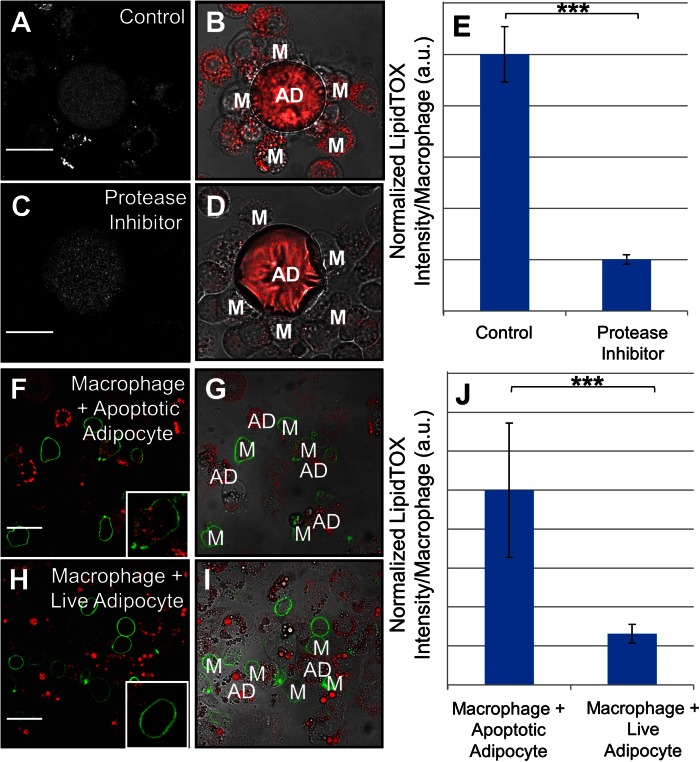
Macrophage interaction with apoptotic adipocytes results in foam cell formation. A–E: Macrophage foam cell formation occurs in the CLS cell culture model (A, B) and is blocked by a broad spectrum cocktail of protease inhibitors (C, D). Primary murine TNF-α-induced apoptotic adipocytes were incubated with J774 cells for 24 h in the absence (A, B) or presence (C, D) of a protease inhibitor cocktail. Samples were labeled with LipidTOX-Red, and the amount of LipidTOX per macrophage quantified in control samples and samples treated with a protease inhibitor cocktail (E). Data are from three independent experiments. F–J: Foam cell formation occurs when macrophages are incubated with apoptotic, but not live, adipocytes. J774 cells were incubated with either TNF-α-induced apoptotic or viable 3T3 L1 adipocytes for 24 h, and their plasma membranes were labeled with Alexa488-CtB (green). Samples were incubated with LipidTOX-Red to label neutral lipid droplets. F, G: When macrophages were incubated with apoptotic adipocytes, numerous LipidTOX-positive macrophages were seen. H, I: Foam cell formation and adipocyte catabolism did not occur when macrophages were incubated with live adipocytes. J: Quantification of the LipidTOX-signal per macrophage in samples with apoptotic adipocytes or live adipocytes. Data are from three independent experiments. Error bars represent the SEM. ***P ≤ 0.001. Size bars 50 μm. AD, adipocyte; M, macrophage.

CLS macrophage lipid accumulation resulting in foam cell formation has been documented in both animal models and human obesity (8, 11). Consistent with these findings, we observed the formation of lipid droplets in the CLS cell culture model (**Fig. 5A, B**). We hypothesized that protease inhibition would prevent the ability of the macrophages to break the adipocyte into small pieces that could be internalized and, thereby, block foam cell formation. To test this, J774 cells interacting with TNF-α-induced apoptotic primary murine adipocytes were incubated for 24 h in the absence (Fig. 5A, B) or presence (Fig. 5C, D) of a cocktail of protease inhibitors with broad specificity. Following co-incubation, cells were fixed and labeled with LipidTOX-Red to detect neutral lipid droplets. In macrophages incubated with apoptotic adipocytes and protease inhibitors, only a small percentage of macrophages contained LipidTOX-labeled droplets (Fig. 5C, D). The amount of LipidTOX per macrophage was quantified for each condition. Incubation with protease inhibitors caused a significant reduction in adipocyte internalization (Fig. 5E). Also, we observed that 60% of macrophages became foam cells after 24 h co-incubation with apoptotic primary murine adipocytes. As a control, phagocytosis of fluorescently labeled beads was quantified in J774 cells in the presence or absence of protease inhibitor cocktail (supplementary Fig. 5B). Treatment with protease inhibitor cocktail did not reduce macrophage phagocytosis of beads (supplementary Fig. 5B). This shows that the effect caused by the protease inhibitor cocktail is due to inhibition of exophagy-mediated uptake.

### Macrophage exophagy is a process specific to dead or dying adipocytes

To determine whether exophagy is a process specific to macrophage interaction with dead adipocytes, J774 cells were incubated for 24 h with either TNF-α-induced apoptotic 3T3 L1 adipocytes (Fig. 5F, G) or viable 3T3 L1 adipocytes (Fig. 5H, I). Following co-incubation, the macrophage plasma membrane was labeled with Alexa488-CtB. Samples were then fixed and labeled with LipidTOX-Red to detect neutral lipid droplets. In macrophages incubated with apoptotic 3T3 L1 adipocytes, 62% of macrophages contained neutral-lipid droplets (inset, Fig. 5F), indicative of foam cell formation. However, in macrophages incubated with viable adipocytes, most macrophages did not contain LipidTOX-labeled droplets (inset, Fig. 5H). The LipidTOX signal per macrophage was quantified for each condition. While foam cell formation was prevalent in macrophages incubated with apoptotic adipocytes, very few macrophages incubated with living adipocytes were LipidTOX positive (Fig. 5J). These data demonstrate that macrophage exophagy is a process specific to dead or dying adipocytes and does not occur when macrophages are in contact with live adipocytes.

## DISCUSSION

In WAT of obese mice, in human tissue, and in a CLS cell culture model, we found that CLS macrophages use F-actin-rich protrusions to create extracellular digestive compartments at the macrophage-adipocyte interface. In the cell culture model, we showed that specialized extracellular compartments (lysosomal synapses) contain lysosomal enzymes, delivered by exocytosis, and they maintain a low pH that is retained by F-actin-rich seals, allowing activity of lysosomal acid hydrolases. Increased lysosome exocytosis occurring during macrophage exophagy in CLSs may be associated with signaling pathways for upregulated lysosome biogenesis, which was recently shown in macrophages contained in inflamed adipose (28). Initial catabolism of the dead adipocyte occurs in the lysosomal synapse, allowing the macrophage to internalize pieces of the adipocyte.

Although the hydrolysis of adipocyte triglycerides was not measured directly, this process may lead to a large uptake of fatty acids, which can be esterified and stored in lipid droplets. Exophagy-mediated foam cell formation is a highly efficient means by which macrophages internalize large amounts of lipid, which may overwhelm the metabolic capacity of the macrophage leading to a maladaptive inflammatory response. Foam cell formation in the model system is blocked by broad spectrum protease inhibitors. The ability to interfere with macrophage exophagy suggests the potential to therapeutically slow the process once the key enzymes and signaling mechanisms are defined.

In our CLS cell culture model, macrophage-like J774 and RAW cells, murine primary BMMs and huMDMs all exhibit similar responses to both apoptotic and pyroptotic 3T3 L1 and primary murine adipocytes, indicating that this is a robust phenomenon. However, it is important to consider that the in vitro work was performed with adipocytes that were already dead, while the in vivo process may involve adipocytes still undergoing apoptosis.

We have shown that macrophages form an extracellular acidic lytic compartment to digest objects that cannot be internalized by standard endocytic mechanisms. We suggest that exophagy is a fundamental process of monocyte-derived cells that occurs in several contexts. A particular example of this biology is osteoclast degradation of bone (16–18). However, there are several notable distinctions between osteoclast catabolism of bone and macrophage catabolism of dead/dying adipocytes. Perhaps most importantly, mesenchymal apoptosis is not required for acute and chronic control of osteoclast-mediated bone turnover in the uninjured mature skeleton. Thus, bone degradation occurs at the matrix surface of a vital tissue, while CLS macrophage biology is triggered by adipocyte injury/death. Thus, the biology detailed herein may be more akin to frustrated phagocytosis, a pro-inflammatory type of macrophage activation characterized by extracellular release of lysosomal contents when the macrophage is facing a target much larger than itself (37, 38). However, in exophagy the macrophages’ ability to catabolize and ingest the adipocyte is not “frustrated.” This apparent link between exophagy and frustrated phagocytosis may provide clues to the interplay between exophagy and WAT inflammation (WATi).

The exophagic process described here may have implications for the pro- and anti-inflammatory aspects of the interaction of macrophages with adipocytes. For instance, does liberation of adipocyte lipid by CLS macrophages promote or prolong the pro-inflammatory macrophage gene expression that characterizes obesity and its complications? The free fatty acids that would be released during exophagy of dead adipocytes have been shown to result in macrophage nuclear factor κB (NF-κB) activation, leading to enhanced secretion of pro-inflammatory chemokines and cytokines (e.g., TNF-α) (7, 39, 40). Thus, exophagy could propagate a feed-forward mechanism of inflammation in which adipocyte death leads to enhanced macrophage secretion of TNF-α, resulting in further adipocyte death and additional cytokine secretion. Alternatively, recent studies suggest that uptake of triacylglycerides via the scavenger receptor CD36 can induce alternative (M2) macrophage activation (41). Thus, it is possible that, similar to macrophage foam cells found in atherosclerotic lesions, macrophage activation in CLSs is not a consequence of lipid accumulation, but rather results from extrinsic mediators (42). We have shown that the extracellular compartments used for exophagy are dynamic (15). They can be tightly sealed at times, but they also transiently open up. This opening can release both catabolic products and lysosomal enzymes into the extracellular space, which might also contribute to WATi. Future studies will focus on understanding the exact nature of the macrophage-adipocyte signaling mechanisms to address these questions and provide a platform for possible therapeutic manipulation of WATi.

## Supplementary Material

Supplemental Data

## References

[1] Hotamisligil G. S. 2006 Inflammation and metabolic disorders. Nature. 444: 860–867. 17167474 10.1038/nature05485

[2] Olefsky J. M., and Glass C. K. 2010 Macrophages, inflammation, and insulin resistance. Annu. Rev. Physiol. 72: 219–246. 20148674 10.1146/annurev-physiol-021909-135846

[3] Lumeng C. N., and Saltiel A. R. 2011 Inflammatory links between obesity and metabolic disease. J. Clin. Invest. 121: 2111–2117. 21633179 10.1172/JCI57132 PMC3104776

[4] Park J., Euhus D. M., and Scherer P. E. 2011 Paracrine and endocrine effects of adipose tissue on cancer development and progression. Endocr. Rev. 32: 550–570. 21642230 10.1210/er.2010-0030 PMC3369575

[5] Wernstedt Asterholm I., Tao C., Morley T. S., Wang Q. A., Delgado-Lopez F., Wang Z. V., and Scherer P. E. 2014 Adipocyte inflammation is essential for healthy adipose tissue expansion and remodeling. Cell Metab. 20: 103–118. 24930973 10.1016/j.cmet.2014.05.005 PMC4079756

[6] Xu H., Barnes G. T., Yang Q., Tan G., Yang D., Chou C. J., Sole J., Nichols A., Ross J. S., Tartaglia L. A., 2003 Chronic inflammation in fat plays a crucial role in the development of obesity-related insulin resistance. J. Clin. Invest. 112: 1821–1830. 14679177 10.1172/JCI19451 PMC296998

[7] Nguyen M. T., Favelyukis S., Nguyen A. K., Reichart D., Scott P. A., Jenn A., Liu-Bryan R., Glass C. K., Neels J. G., and Olefsky J. M. 2007 A subpopulation of macrophages infiltrates hypertrophic adipose tissue and is activated by free fatty acids via Toll-like receptors 2 and 4 and JNK-dependent pathways. J. Biol. Chem. 282: 35279–35292. 17916553 10.1074/jbc.M706762200

[8] Lumeng C. N., Deyoung S. M., Bodzin J. L., and Saltiel A. R. 2007 Increased inflammatory properties of adipose tissue macrophages recruited during diet-induced obesity. Diabetes. 56: 16–23. 17192460 10.2337/db06-1076

[9] Cancello R., Henegar C., Viguerie N., Taleb S., Poitou C., Rouault C., Coupaye M., Pelloux V., Hugol D., Bouillot J. L., 2005 Reduction of macrophage infiltration and chemoattractant gene expression changes in white adipose tissue of morbidly obese subjects after surgery-induced weight loss. Diabetes. 54: 2277–2286. 16046292 10.2337/diabetes.54.8.2277

[10] Cinti S., Mitchell G., Barbatelli G., Murano I., Ceresi E., Faloia E., Wang S., Fortier M., Greenberg A. S., and Obin M. S. 2005 Adipocyte death defines macrophage localization and function in adipose tissue of obese mice and humans. J. Lipid Res. 46: 2347–2355. 16150820 10.1194/jlr.M500294-JLR200

[11] Shapiro H., Pecht T., Shaco-Levy R., Harman-Boehm I., Kirshtein B., Kuperman Y., Chen A., Bluher M., Shai I., and Rudich A. 2013 Adipose tissue foam cells are present in human obesity. J. Clin. Endocrinol. Metab. 98: 1173–1181. 23372170 10.1210/jc.2012-2745

[12] Giordano A., Murano I., Mondini E., Perugini J., Smorlesi A., Severi I., Barazzoni R., Scherer P. E., and Cinti S. 2013 Obese adipocytes show ultrastructural features of stressed cells and die of pyroptosis. J. Lipid Res. 54: 2423–2436. 23836106 10.1194/jlr.M038638 PMC3735940

[13] Zhang W. Y., Gaynor P. M., and Kruth H. S. 1997 Aggregated low density lipoprotein induces and enters surface-connected compartments of human monocyte-macrophages. Uptake occurs independently of the low density lipoprotein receptor. J. Biol. Chem. 272: 31700–31706. 9395512 10.1074/jbc.272.50.31700

[14] Grosheva I., Haka A. S., Qin C., Pierini L. M., and Maxfield F. R. 2009 Aggregated LDL in contact with macrophages induces local increases in free cholesterol levels that regulate local actin polymerization. Arterioscler. Thromb. Vasc. Biol. 29: 1615–1621. 19556523 10.1161/ATVBAHA.109.191882 PMC2755184

[15] Haka A. S., Grosheva I., Chiang E., Buxbaum A. R., Baird B. A., Pierini L. M., and Maxfield F. R. 2009 Macrophages create an acidic extracellular hydrolytic compartment to digest aggregated lipoproteins. Mol. Biol. Cell. 20: 4932–4940. 19812252 10.1091/mbc.E09-07-0559 PMC2785736

[16] Baron R., Neff L., Louvard D., and Courtoy P. J. 1985 Cell-mediated extracellular acidification and bone resorption: evidence for a low pH in resorbing lacunae and localization of a 100-kD lysosomal membrane protein at the osteoclast ruffled border. J. Cell Biol. 101: 2210–2222. 3905822 10.1083/jcb.101.6.2210 PMC2114017

[17] Stenbeck G. 2002 Formation and function of the ruffled border in osteoclasts. Semin. Cell Dev. Biol. 13: 285–292. 12243728 10.1016/s1084952102000587

[18] Jurdic P., Saltel F., Chabadel A., and Destaing O. 2006 Podosome and sealing zone: specificity of the osteoclast model. Eur. J. Cell Biol. 85: 195–202. 16546562 10.1016/j.ejcb.2005.09.008

[19] Moore K. J., and Tabas I. 2011 Macrophages in the pathogenesis of atherosclerosis. Cell. 145: 341–355. 21529710 10.1016/j.cell.2011.04.005 PMC3111065

[20] Hong J., Stubbins R. E., Smith R. R., Harvey A. E., and Nunez N. P. 2009 Differential susceptibility to obesity between male, female and ovariectomized female mice. Nutr. J. 8: 11. 19220919 10.1186/1475-2891-8-11 PMC2650703

[21] Motoshima H., Wu X., Sinha M. K., Hardy V. E., Rosato E. L., Barbot D. J., Rosato F. E., and Goldstein B. J. 2002 Differential regulation of adiponectin secretion from cultured human omental and subcutaneous adipocytes: effects of insulin and rosiglitazone. J. Clin. Endocrinol. Metab. 87: 5662–5667. 12466369 10.1210/jc.2002-020635

[22] Frost S. C., and Lane M. D. 1985 Evidence for the involvement of vicinal sulfhydryl groups in insulin-activated hexose transport by 3T3-L1 adipocytes. J. Biol. Chem. 260: 2646–2652. 3882699

[23] Lin J., Page K. A., Della-Fera M. A., and Baile C. A. 2004 Evaluation of adipocyte apoptosis by laser scanning cytometry. Int. J. Obes. Relat. Metab. Disord. 28: 1535–1540. 15356667 10.1038/sj.ijo.0802777

[24] Sagulenko V., Thygesen S. J., Sester D. P., Idris A., Cridland J. A., Vajjhala P. R., Roberts T. L., Schroder K., Vince J. E., Hill J. M., 2013 AIM2 and NLRP3 inflammasomes activate both apoptotic and pyroptotic death pathways via ASC. Cell Death Differ. 20: 1149–1160. 23645208 10.1038/cdd.2013.37 PMC3741496

[25] Huynh K. K., Eskelinen E. L., Scott C. C., Malevanets A., Saftig P., and Grinstein S. 2007 LAMP proteins are required for fusion of lysosomes with phagosomes. EMBO J. 26: 313–324. 17245426 10.1038/sj.emboj.7601511 PMC1783450

[26] Harter C., and Mellman I. 1992 Transport of the lysosomal membrane glycoprotein lgp120 (lgp-A) to lysosomes does not require appearance on the plasma membrane. J. Cell Biol. 117: 311–325. 1560028 10.1083/jcb.117.2.311 PMC2289424

[27] Colvin R. A., Means T. K., Diefenbach T. J., Moita L. F., Friday R. P., Sever S., Campanella G. S., Abrazinski T., Manice L. A., Moita C., 2010 Synaptotagmin-mediated vesicle fusion regulates cell migration. Nat. Immunol. 11: 495–502. 20473299 10.1038/ni.1878 PMC2951881

[28] Xu X., Grijalva A., Skowronski A., van Eijk M., Serlie M. J., and Ferrante A. W. Jr 2013 Obesity activates a program of lysosomal-dependent lipid metabolism in adipose tissue macrophages independently of classic activation. Cell Metab. 18: 816–830. 24315368 10.1016/j.cmet.2013.11.001 PMC3939841

[29] Hotamisligil G. S., Shargill N. S., and Spiegelman B. M. 1993 Adipose expression of tumor necrosis factor-alpha: direct role in obesity-linked insulin resistance. Science. 259: 87–91. 7678183 10.1126/science.7678183

[30] Zhang Y., and Huang C. 2012 Targeting adipocyte apoptosis: a novel strategy for obesity therapy. Biochem. Biophys. Res. Commun. 417: 1–4. 22172945 10.1016/j.bbrc.2011.11.158

[31] Bennett G., Strissel K. J., DeFuria J., Wang J., Wu D., Burkly L. C., and Obin M. S. 2014 Deletion of TNF-like weak inducer of apoptosis (TWEAK) protects mice from adipose and systemic impacts of severe obesity. Obesity (Silver Spring). 22: 1485–1494. 24616441 10.1002/oby.20726 PMC4283503

[32] Singh R. K., Barbosa-Lorenzi V. C., Lund F. W., Grosheva I., Maxfield F. R., and Haka A. S. 2016 Degradation of aggregated LDL occurs in complex extracellular sub-compartments of the lysosomal synapse. J. Cell Sci. 129: 1072–1082. 26801085 10.1242/jcs.181743 PMC4813320

[33] Mayor S., Rothberg K. G., and Maxfield F. R. 1994 Sequestration of GPI-anchored proteins in caveolae triggered by cross-linking. Science. 264: 1948–1951. 7516582 10.1126/science.7516582

[34] Beletskii A., Cooper M., Sriraman P., Chiriac C., Zhao L., Abbot S., and Yu L. 2005 High-throughput phagocytosis assay utilizing a pH-sensitive fluorescent dye. Biotechniques. 39: 894–897. 16382909 10.2144/000112001

[35] Grinstein S., Nanda A., Lukacs G., and Rotstein O. 1992 V-ATPases in phagocytic cells. J. Exp. Biol. 172: 179–192. 1491224 10.1242/jeb.172.1.179

[36] Bowman B. J., and Bowman E. J. 2002 Mutations in subunit C of the vacuolar ATPase confer resistance to bafilomycin and identify a conserved antibiotic binding site. J. Biol. Chem. 277: 3965–3972. 11724795 10.1074/jbc.M109756200

[37] Labrousse A. M., Meunier E., Record J., Labernadie A., Beduer A., Vieu C., Ben Safta T., and Maridonneau-Parini I. 2011 Frustrated phagocytosis on micro-patterned immune complexes to characterize lysosome movements in live macrophages. Front. Immunol. 2: 51. 22566841 10.3389/fimmu.2011.00051 PMC3341964

[38] Singh A. V., Batuwangala M., Mundra R., Mehta K., Patke S., Falletta E., Patil R., and Gade W. N. 2014 Biomineralized anisotropic gold microplate-macrophage interactions reveal frustrated phagocytosis-like phenomenon: a novel paclitaxel drug delivery vehicle. ACS Appl. Mater. Interfaces. 6: 14679–14689. 25046687 10.1021/am504051b

[39] Lee J. Y., Ye J., Gao Z., Youn H. S., Lee W. H., Zhao L., Sizemore N., and Hwang D. H. 2003 Reciprocal modulation of Toll-like receptor-4 signaling pathways involving MyD88 and phosphatidylinositol 3-kinase/AKT by saturated and polyunsaturated fatty acids. J. Biol. Chem. 278: 37041–37051. 12865424 10.1074/jbc.M305213200

[40] Lee J. Y., Sohn K. H., Rhee S. H., and Hwang D. 2001 Saturated fatty acids, but not unsaturated fatty acids, induce the expression of cyclooxygenase-2 mediated through Toll-like receptor 4. J. Biol. Chem. 276: 16683–16689. 11278967 10.1074/jbc.M011695200

[41] Huang S. C., Everts B., Ivanova Y., O’Sullivan D., Nascimento M., Smith A. M., Beatty W., Love-Gregory L., Lam W. Y., O’Neill C. M., 2014 Cell-intrinsic lysosomal lipolysis is essential for alternative activation of macrophages. Nat. Immunol. 15: 846–855. 25086775 10.1038/ni.2956 PMC4139419

[42] Spann N. J., Garmire L. X., McDonald J. G., Myers D. S., Milne S. B., Shibata N., Reichart D., Fox J. N., Shaked I., Heudobler D., 2012 Regulated accumulation of desmosterol integrates macrophage lipid metabolism and inflammatory responses. Cell. 151: 138–152. 23021221 10.1016/j.cell.2012.06.054 PMC3464914

