# Supplementary material for: Exocytosis of macrophage lysosomes leads to digestion of apoptotic adipocytes and foam cell formation
Source: J Lipid Res. 2016 Jun;57(6):980–92. doi: 10.1194/jlr.M064089 (PMC4878183; doi:10.1194/jlr.M064089)
Supplement: Supplemental Data [file supp_57_6_980__index.html]

Exocytosis of Macrophage Lysosomes Leads to Digestion of Apoptotic Adipocytes and Foam Cell Formation — Exocytosis of macrophage lysosomes leads to digestion of apoptotic adipocytes and foam cell formation — Supplemental Data 

# Exocytosis of macrophage lysosomes leads to digestion of apoptotic adipocytes and foam cell formation

## Supplemental Data

- Supplemental Figures (.pdf, 877 KB) - Supplementary figures and figure legends.
- Supplemental Movie 1 (.mov, 1.4 MB) - Supplemental Movie 1. Time-lapse ratiometric live cell imaging reveals a neutral pH at regions of interaction between macrophages and live adipocytes.
- Supplemental Movie 2 (.mov, 1.0 MB) - Supplemental Movie 2. Time-lapse ratiometric live cell imaging reveals dynamics of extracellular compartments.
- Supplemental Movie 3 (.mov, 4.9 MB) - Supplemental Movie 3. FIB-SEM reveals extracellular compartments at the macrophage apoptotic-adipocyte interface that can hold a pH gradient.
- Supplemental Movie 4 (.mov, 15.4 MB) - Supplemental Movie 4. 3D visualization of extracellular compartments at the macrophage-apoptotic adipocyte interface that are sealed.
