# Supplementary material for: Exocytosis of macrophage lysosomes leads to digestion of apoptotic adipocytes and foam cell formation
Source: J Lipid Res. 2016 Jun;57(6):980–92. doi: 10.1194/jlr.M064089 (PMC4878183; doi:10.1194/jlr.M064089)

Supplementary Figure S I. Illustration of data analysis for in vivo macrophage surface LAMP-1 quantification. Macrophages were identified using an F4/80 primary antibody and an FITC secondary antibody **(A)**. Permeabilized cells were identified using a calnexin antibody and a Cy5 secondary antibody **(B)**. LAMP-1 was labeled with an anti-LAMP-1 antibody and a Cy3 secondary antibody **(C),** and cell nuclei were labeled with DAPI **(D)**. A binary mask was created using the FITC signal intensity to select F4/80 positive macrophages **(E)**. This image was multiplied pixel-by-pixel with an inverted binary mask created using the Cy5 signal, to select Calnexin-negative non-permeabilized cells **(F)**. (Permeabilized macrophages that were excluded from the analysis are highlighted by arrows.) The resultant mask **(G and G')** was then applied to the Cy3 channel **(H)** to display LAMP-1 signals from non-permeabilized macrophages (F4/80 positive and calnexin negative) **(I)**. The fluorescence intensity of surface LAMP-1 labeling **(I)** was increased 10 fold for better visualization. The surface LAMP-1 signal in each image was divided by the number of non-permeabilized cells in that field, as determined by DAPI staining, to generate an average value for plasma membrane LAMP-1 per cell.

Supplementary Figure S II. Abundance of LAMP-1 in CLS and resident macrophages in murine WAT and in human monocyte derived macrophages. WAT from mice on the HFD were permeabilized with Triton and labeled with antibodies to **(A)** LAMP-1, **(B)** F4/80 and **(C)** DAPI. An enlarged view of a permeabilized macrophage showing LAMP-1 labeling of lysosomes is included as an inset (B). WAT from mice on the LFD were also permeabilized with Triton and labeled with antibodies to **(D)** LAMP-1, **(E)** F4/80 and **(F)** DAPI. **(G)** Lysosome LAMP-1 levels were quantified from CLS macrophages (A-C) from five mice on the HFD and resident macrophages (D-F) from five mice on the LFD. There was a trend toward greater lysosomal LAMP-1 in detergent-permeabilized CLS macrophages but it was not statistically significant. Error bars represent the SEM. Not significant (n.s.) student's *t* test. **(H)** Quantification of the macrophage surface levels of LAMP-1 in huMDMs incubated with UV-induced apoptotic primary murine adipocytes compared to control huMDMs. Data are from 548 cells in one experiment. Error bar represents the SEM. *** $P \leq 0.001$ student's *t* test. **(I)** Merged images of Alexa488 and phase contrast from co-cultures of J774 macrophages and TNF-α induced apoptotic or viable 3T3 L1 adipocytes that were immunostained with an irrelevant specificity antibody, followed by incubation with a secondary antibody conjugated with AlexaFluor488. Representative adipocytes (AD) and macrophages (M) are labeled.

Supplementary Figure S III. Examples of lysosomal synapses that are not penetrated by biotin-Alexa546. J774 macrophages were incubated overnight with biotin-fluorescein-dextran to deliver the dextran to lysosomes. Cells were then incubated with streptavidin-labeled apoptotic 3T3 L1 adipocytes (UV treated) for 90 min followed by a 30 sec treatment with 200 µM biotin-Alexa546 to mark extracellular streptavidin labeled structures. Next, 200 µM biotin was applied for 10 min to block any remaining free streptavidin. Cells were then fixed and permeabilized to remove unbound biotin-fluorescein-dextran from the lysosomes. Representative images of biotin-fluorescein-dextran **(A)**, biotin-Alexa546 **(B)** and merged images superimposed on phase contrast image **(C)**. Arrows indicate regions of lysosomal exocytosis that do not co-localize with biotin-Alexa546 (green, shown in detail in the inset).

Supplementary Figure S IV. Cell death induced by pyroptosis leads to macrophage lysosome exocytosis, but it does not occur in macrophages incubated with viable adipocytes. (A-C) RAW264.7 macrophages were incubated overnight with biotin-fluorescein-dextran to deliver the dextran to lysosomes. 3T3 L1 adipocytes were incubated with 10 ng/ml lipopolysaccharide for 4 hr followed by a 2 hr incubation with 10 µM nigericin to induce pyroptosis. The adipocytes were rinsed extensively. Macrophages were then incubated with streptavidin-labeled pyroptotic adipocytes for 90 min followed by a 30 sec treatment with 200

μM biotin-Alexa546 to mark extracellular streptavidin labeled structures. Next, 200 μM biotin was applied for 10 min to block any remaining free streptavidin. Cells were then fixed and permeabilized to remove unbound biotin-fluorescein-dextran from the lysosomes. Representative image of biotin-fluorescein-dextran **(A)**, biotin-Alexa546 **(B)** and a merged image superimposed on phase contrast image **(C)**. Co-localization of the fluorescein and Alexa546 signals (yellow) demonstrates that lysosomal contents are delivered to extracellular compartments (arrow and inset). **(D-E)** J774 cells were incubated overnight with biotin-fluorescein-dextran to deliver the dextran to lysosomes. Cells were then incubated with either TNF-α induced apoptotic (D) or streptavidin-labeled viable (E) 3T3 L1 adipocytes for 90 min. Cells were then fixed and permeabilized to remove unbound biotin-fluorescein-dextran from the lysosomes. **(F)** Quantification of the intensity of biotin-fluorescein bound to streptavidin-labeled adipocytes. Data are from three independent experiments. Error bar represents the SEM. *** $P \leq 0.001$ student's *t* test. **(G)** Percentage of positive propidium iodide stained 3T3 L1 adipocytes after having their surface labeled with NHS-Biotin (DMSO diluted) followed by streptavidin; UV-irradiated and 25 nM TNF-α treated adipocytes were used as a positive control of cell death. **(H)** J774 macrophages were incubated with live 3T3 L1 adipocytes labeled with CypHer 5E, a pH sensitive fluorophore, and Alexa488, a pH insensitive fluorophore. Ratiometric image shows regions at the interface between macrophages and the adipocytes that present a neutral pH.

Supplementary Figure S V. Macrophage phagocytosis is not affected by bafilomycin or protease inhibitor treatment. J774 cells were incubated with Fluoresbrite YG latex beads for 90 min in the presence or absence of 2 μM bafilomycin A1 **(A)**, or in the presence or absence of protease inhibitor cocktail, with broad specificity for the inhibition of serine, cysteine, aspartic and aminopeptidases (1:800 dilution) **(B)**. Neither bafilomycin A1 nor protease inhibitor cocktail inhibit macrophage phagocytosis of beads. Error bars represents the SEM. n.s. not significant.

Supplementary Movie S I. *Time-lapse ratiometric live cell imaging reveals a neutral pH at regions of interaction between macrophages and live adipocytes.* J774 cells were incubated for 30 min with live 3T3 L1 adipocytes labeled with CypHer 5E, a pH sensitive fluorophore, and Alexa488, a pH insensitive fluorophore. The pH value within each pixel was determined by comparison with ratio images obtained in calibration buffers. The data set was acquired for 20 min with 1 min between frames.

Supplementary Movie S II. *Time-lapse ratiometric live cell imaging reveals dynamics of extracellular compartments.* J774 cells were incubated for 30 min with UV-induced apoptotic 3T3 L1 adipocytes labeled with CypHer 5E, a pH sensitive fluorophore, and Alexa488, a pH insensitive fluorophore. The pH value within each pixel was determined by comparison with ratio images obtained in calibration buffers. The data set was acquired for 20 min with 1 min between frames. The acidic regions are transient, dissipating and reforming as the sub-compartments formed at the macrophage-adipocyte interface open and close. Some portions of the adipocyte were internalized and can be seen moving as independent vesicles.

Supplementary Movie S III. *FIB-SEM reveals extracellular compartments at the macrophage apoptotic-adipocyte interface that can hold a pH gradient.* The entire FIB-SEM z-stack with the reconstructed compartments overlaid on the x y plane in their respective colors.

Supplementary Movie S IV. *3D visualization of extracellular compartments at the macrophage-apoptotic adipocyte interface that are sealed.* The FIB-SEM data is varied in the z dimension with a three dimensional reconstruction of the compartments overlaid followed by 3D visualization of both the cells and compartments used for exophagy.

Supplementary Figure S I. Illustration of data analysis for in vivo macrophage surface LAMP-1 quantification

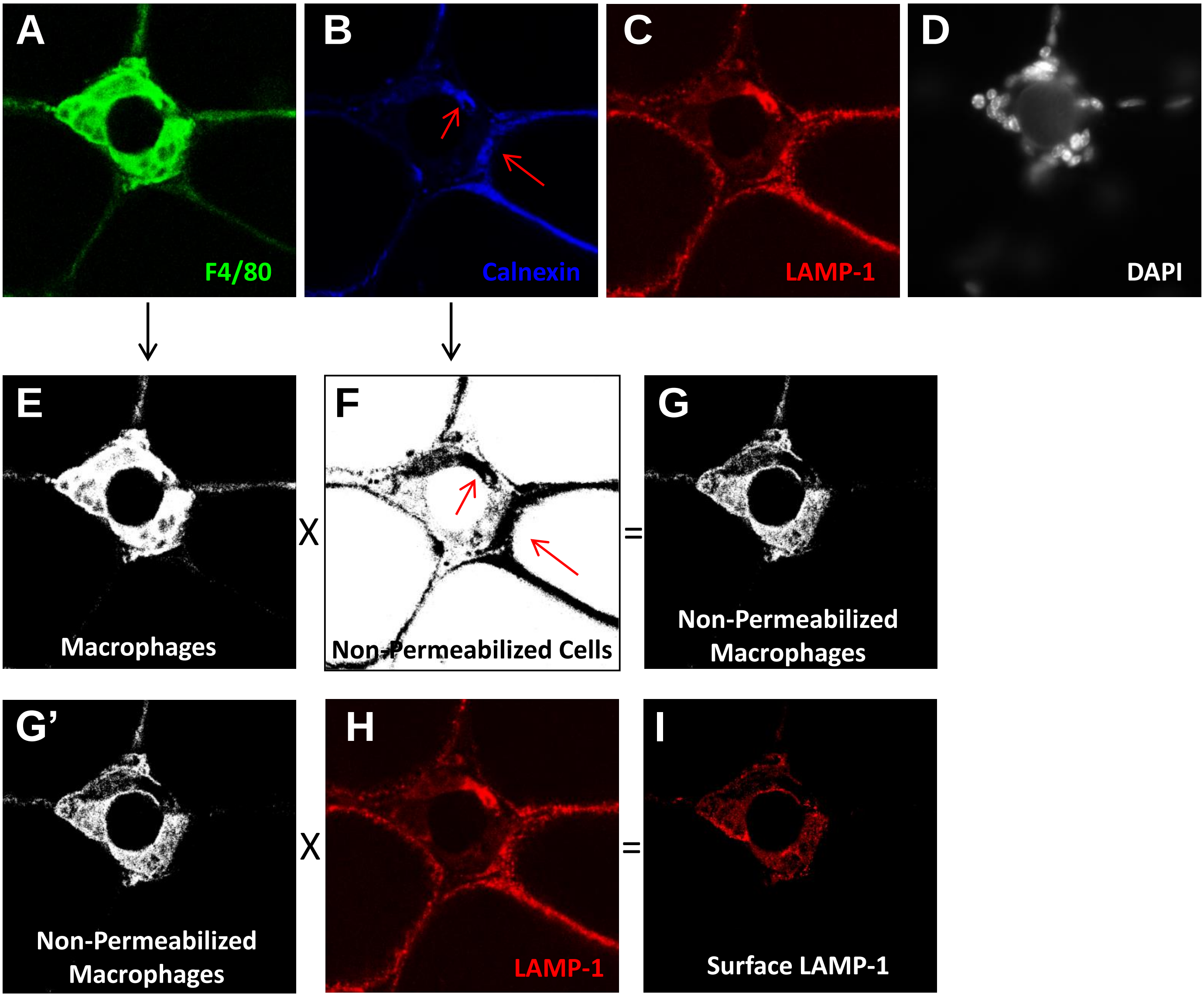

Supplementary Figure S II. Abundance of LAMP-1 in CLS and resident macrophages in murine WAT and in human monocyte derived macrophages.

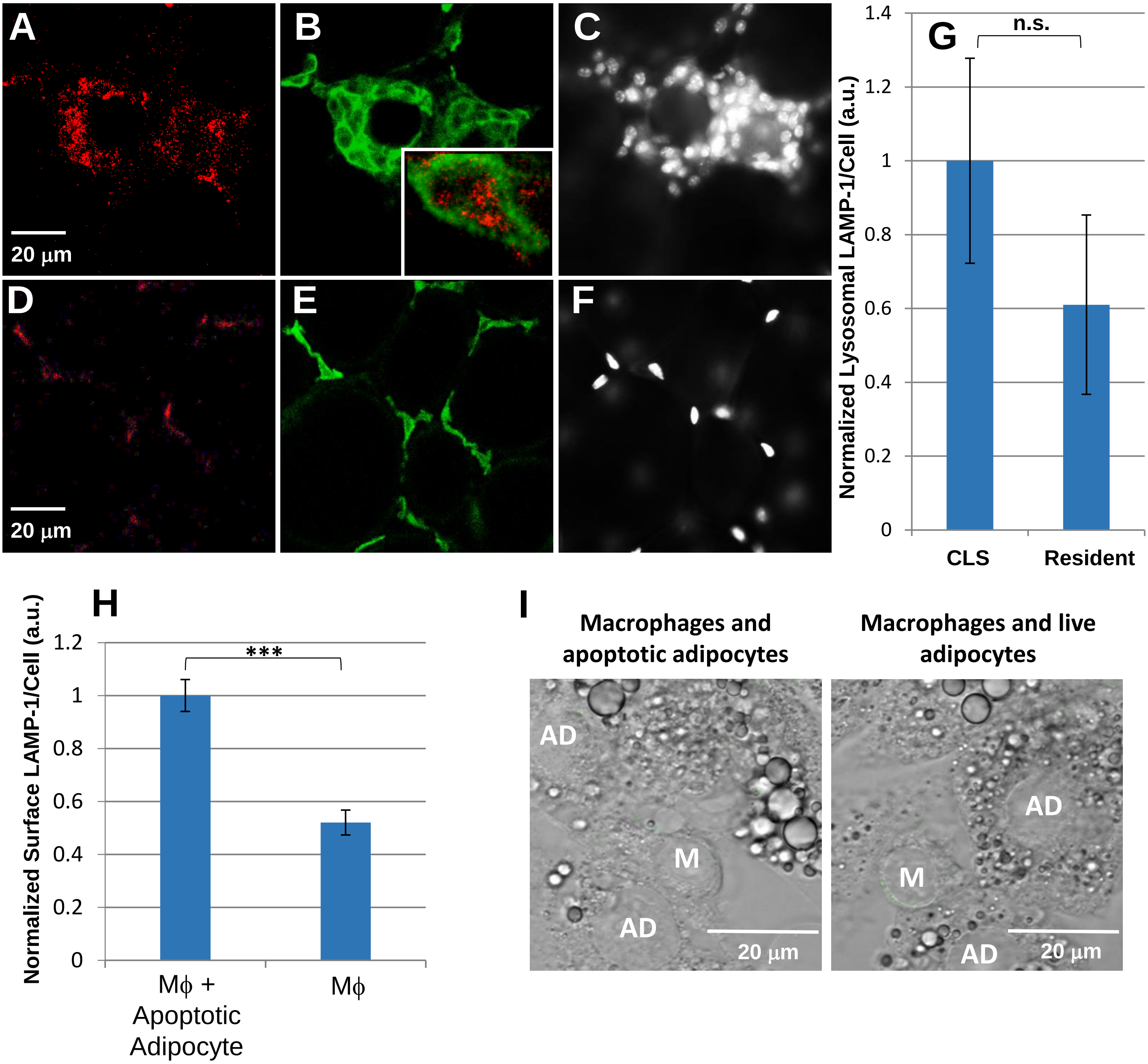

Supplementary Figure S III. Examples of lysosomal synapses that are not penetrated by biotin-Alexa546

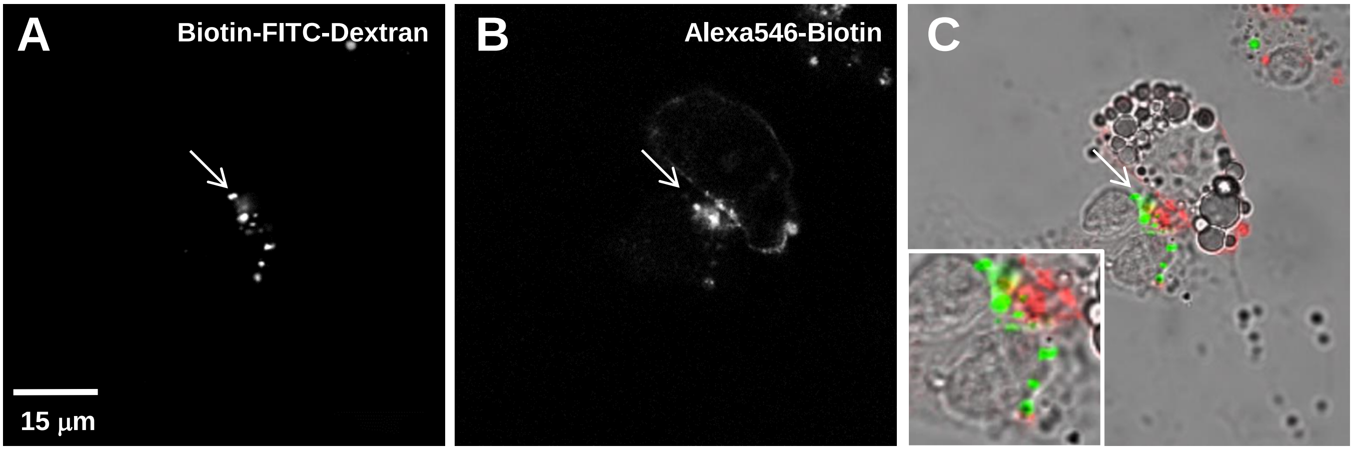

Supplementary Figure S IV. Cell death induced by pyroptosis leads to lysosome exocytosis, but it does not occur in macrophages incubated with viable adipocytes.

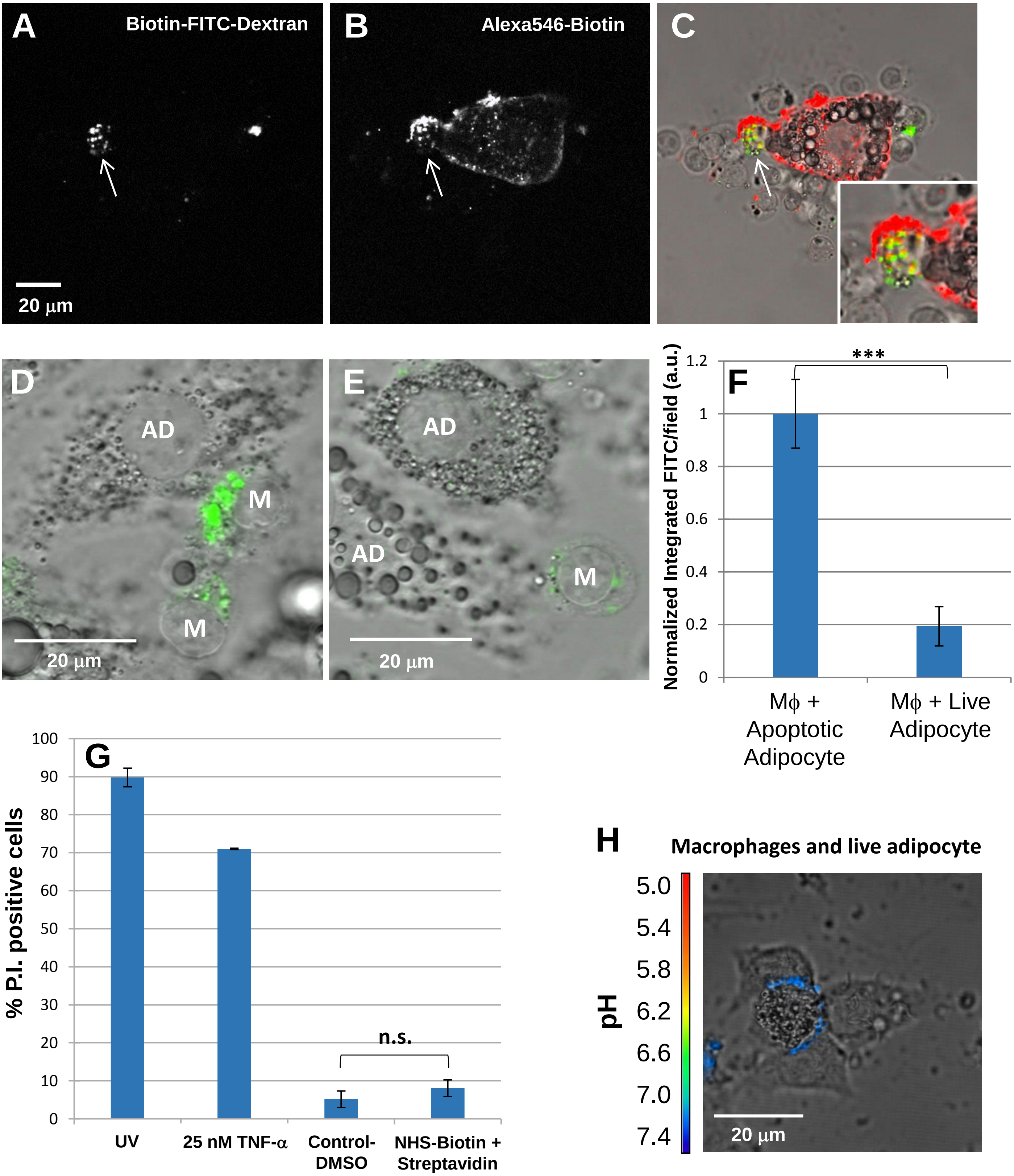

Supplementary Figure S V. Macrophage phagocytosis not effected by bafilomycin or protease inhibitor treatment

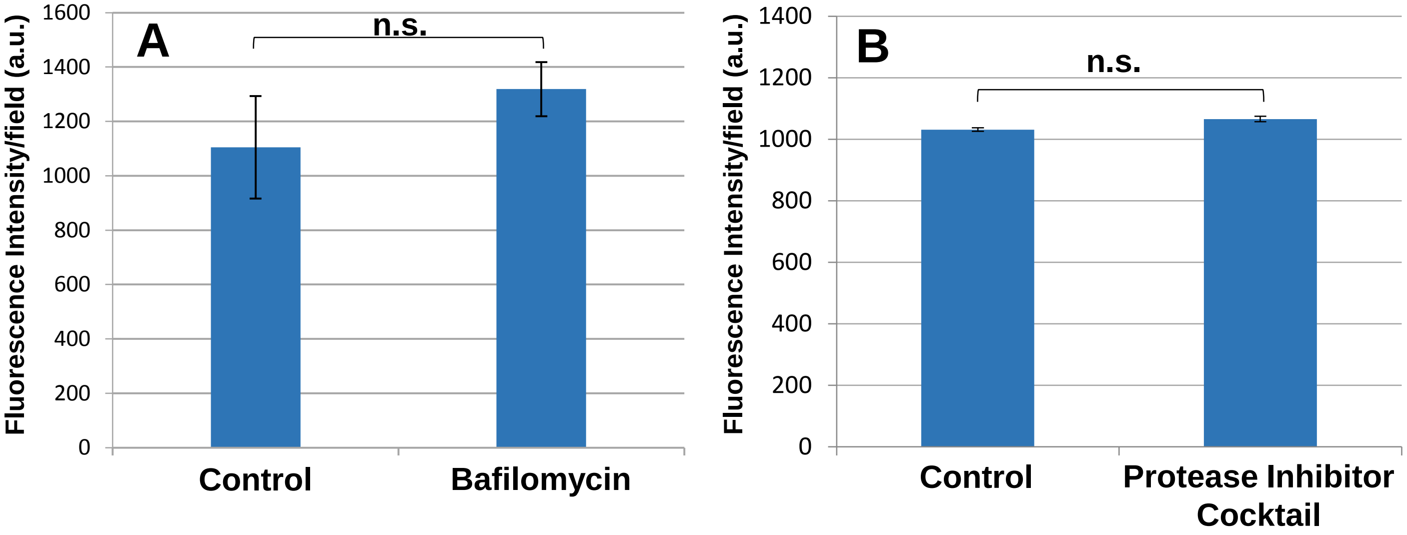

Supplement: Supplemental Data [file 10.1194_M064089_jlr.M064089-1.pdf]
